# The Immediate and Short-Term Effects of Transcutaneous Spinal Cord Stimulation and Peripheral Nerve Stimulation on Corticospinal Excitability

**DOI:** 10.3389/fnins.2021.749042

**Published:** 2021-10-21

**Authors:** Yazi Al’joboori, Ricci Hannah, Francesca Lenham, Pia Borgas, Charlotte J. P. Kremers, Karen L. Bunday, John Rothwell, Lynsey D. Duffell

**Affiliations:** ^1^Department of Medical Physics & Biomedical Engineering, University College London, London, United Kingdom; ^2^Sobell Department of Motor Neuroscience and Movement Disorders, Institute of Neurology, University College London, London, United Kingdom; ^3^Psychology, School of Social Sciences, University of Westminster, London, United Kingdom

**Keywords:** corticospinal excitability, paired associative stimulation (PAS), peripheral nerve stimulation (PNS), rehabilitation, spinal cord stimulation (SCS), transcranial magnetic stimulation

## Abstract

Rehabilitative interventions involving electrical stimulation show promise for neuroplastic recovery in people living with Spinal Cord Injury (SCI). However, the understanding of how stimulation interacts with descending and spinal excitability remain unclear. In this study we compared the immediate and short-term (within a few minutes) effects of pairing Transcranial Magnetic Stimulation (TMS) with transcutaneous Spinal Cord stimulation (tSCS) and Peripheral Nerve Stimulation (PNS) on Corticospinal excitability in healthy subjects. Three separate experimental conditions were assessed. In Experiment I, paired associative stimulation (PAS) was applied, involving repeated pairing of single pulses of TMS and tSCS, either arriving simultaneously at the spinal motoneurones (PAS_0ms_) or slightly delayed (PAS_5ms_). Corticospinal and spinal excitability, and motor performance, were assessed before and after the PAS interventions in 24 subjects. Experiment II compared the immediate effects of tSCS and PNS on corticospinal excitability in 20 subjects. Experiment III compared the immediate effects of tSCS with tSCS delivered at the same stimulation amplitude but modulated with a carrier frequency (in the kHz range) on corticospinal excitability in 10 subjects. Electromyography (EMG) electrodes were placed over the Tibialis Anterior (TA) soleus (SOL) and vastus medialis (VM) muscles and stimulation electrodes (cathodes) were placed on the lumbar spine (tSCS) and lateral to the popliteal fossa (PNS). TMS over the primary motor cortex (M1) was paired with tSCS or PNS to produce Motor Evoked Potentials (MEPs) in the TA and SOL muscles. Simultaneous delivery of repetitive PAS (PAS_0ms_) increased corticospinal excitability and H-reflex amplitude at least 5 min after the intervention, and dorsiflexion force was increased in a force-matching task. When comparing effects on descending excitability between tSCS and PNS, a subsequent facilitation in MEPs was observed following tSCS at 30-50 ms which was not present following PNS. To a lesser extent this facilitatory effect was also observed with HF- tSCS at subthreshold currents. Here we have shown that repeated pairing of TMS and tSCS can increase corticospinal excitability when timed to arrive simultaneously at the alpha-motoneurone and can influence functional motor output. These results may be useful in optimizing stimulation parameters for neuroplasticity in people living with SCI.

## Introduction

Non-invasive electrical stimulation (ES) is commonly used alongside activity-based rehabilitation to improve or recover motor function in people with Spinal Cord Injury (SCI). Recent studies have shown that non-invasive ES applied over the thoracolumbar cord (transcutaneous spinal cord stimulation tSCS) enabled *immediate* restoration of some voluntary movement in people with complete and incomplete SCI (Gerasimenko et al., 2015a; Hofstoetter et al., 2015). These effects appear to improve over several weeks or months of tSCS combined with volitional movement (Gerasimenko et al., 2015a; Sayenko et al., 2019). As these observations were made when tSCS is delivered simultaneously with volitional functional movements (such as walking) over a long duration, we should consider immediate, short- and long-term effects independently. The underlying mechanisms of tSCS on motor output are not yet well understood. tSCS is believed to activate large-to-medium diameter sensory fibers (particularly Ia afferent fibers) within the posterior roots (Rattay et al., 2000), capable of producing trans-synaptic activation of α-motoneurons (posterior root-muscle reflexes, PRRs) (Hofstoetter et al., 2018, 2019). It has been suggested that immediate effects on voluntary motor performance could be due to temporal summation between the afferent input and voluntary descending commands from spared pathways (Gerasimenko et al., 2015c). Meanwhile, short-term effects could be due to spike-timing dependent plasticity (STDP) like mechanisms, where repeated pairing of descending and ascending input cause strengthening of synaptic connections in the cord (cortico-motoneuronal and/or Ia-afferent-α-motoneuron) (Nishimura et al., 2013).

Our aim was to examine the plausibility of two potential mechanisms in healthy humans: i) that repeated pairing of descending and afferent volleys at the spinal cord produces lasting changes in corticospinal excitability and voluntary motor performance, and; ii) that afferent input [30 Hz trains of tSCS or peripheral nerve stimulation (PNS)] has immediate effects on the excitability of corticospinal pathways. Our approach was to adopt a highly-controlled, albeit somewhat artificial, set-up involving the pairing of single motor cortex and spinal cord stimuli, via transcranial magnetic stimulation (TMS) and tSCS. This approach benefits from tight control over the timing, number and frequency of ascending and descending volleys that would not otherwise be possible under conditions of volitional movement. Previous studies that have used this paired associative stimulation (PAS) approach, have paired TMS with PNS rather than tSCS. Those studies have observed corticospinal facilitation when the two inputs are timed to coincide at a cortical (Stefan et al., 2000, 2002; Ridding and Uy, 2003; Mrachacz-Kersting et al., 2007; Roy et al., 2007) or spinal (Taylor and Martin, 2009; Bunday and Perez, 2012) level. Here we have paired TMS with tSCS, with the main difference being the activation of several posterior roots with tSCS, compared with the backfiring of α-motoneurons in a single mixed nerve with PNS.

In our first experiment, we adapted a PAS paradigm, thought to rely on STDP-like mechanisms, that had previously been used in rodents (Mishra et al., 2017). Their study confirmed that 100 pairs of stimuli, timed to coincide in the spinal cord, produced a lasting effect (up to 40 min) on responses to cortical and spinal stimulation, thus indicating enhanced spinal excitability. Unfortunately, they did not examine the effects on voluntary motor performance and so it is not clear whether the excitability changes were functionally relevant, and their experiment was not done in humans.

Early PAS studies targeting the spinal cord in humans, which used PNS, relied on the backfiring of α-motoneurons as the conditioning stimulus (Taylor and Martin, 2009; Bunday and Perez, 2012). When the two inputs coincided at a spinal level, H-reflex amplitude was either facilitated (Cortes et al., 2011) or unaffected (Leukel et al., 2012), and H-Reflexes conditioned by both cortical and cervicomedullary stimulation were increased, suggesting that (at least some) neural plasticity was induced within the spinal cord (Leukel et al., 2012). One group has reported that PAS with a high-frequency peripheral component (0.2 Hz TMS paired with 100Hz PNS, timed to coincide at the spinal cord level) enhanced motor output in healthy subjects (Shulga et al., 2016; Mezes et al., 2020) and in pilot studies of people living with SCI (Tolmacheva et al., 2017, 2019; Rodionov et al., 2020; Shulga et al., 2020). However, as a therapeutic tool, this is limited by the relatively few spinal segments, and therefore muscles, that are targeted by the PNS approach. By contrast, tSCS targets multiple segments, evidenced by the elicitation of PRRs in multiple bilateral muscles at the same time (Minassian et al., 2007). Human PAS studies incorporating tSCS have found the immediate interaction effects to be muscle and muscle activity dependent (Roy et al., 2014). Currently, only a few studies have examined the after-effects of a PAS protocol incorporating TMS and tSCS in humans. One study, where the two inputs were timed to coincide at a spinal level, found decreased corticospinal and increased spinal excitability following 40 min of PAS (Dixon et al., 2016). Another study found that PAS combined with locomotor training modulated spinal excitability during locomotion, which was dependent on the timing of the PAS inputs (Pulverenti et al., 2021). These studies however, did not examine whether these changes lead to any improvements in motor performance.

Given that epidural SCS has been observed to have instantaneous beneficial effects on motor output in people with SCI (Angeli et al., 2014), we additionally explored the effects of short trains of tSCS, delivered in different locations and with different waveforms. This was done in healthy participants, to provide insight about the optimal parameters and possible underlying mechanisms of the effects that have been observed after SCI. Since any changes in excitability could potentially contribute to the immediate improvements in motor function, we adopted a condition-test approach, whereby we assessed the short-term (<1 s) effects of tSCS or PNS stimuli on corticospinal excitability, by delivering TMS shortly after the stimuli. tSCS, when used therapeutically in people living with SCI, is typically delivered at between 15 and 30 Hz (Hofstoetter et al., 2013, 2015; Al’joboori et al., 2020) or in ultra-high frequency (HF) bursts (10 kHz bursts delivered at 15-30 Hz), which are thought to minimize the pain and discomfort of tSCS delivered with traditional waveforms (Gerasimenko et al., 2015a, b; Gad et al., 2017; Sayenko et al., 2019). Therefore, we examined the immediate effects of brief 30Hz trains of stimuli. We had two questions. Firstly, what are the immediate effects of short 30 Hz trains of tSCS compared to PNS on corticospinal excitability. Secondly, what are the immediate effects of short 30 Hz trains of tSCS delivered with traditional waveforms compared to high-frequency (10 kHz) bursts. These experiments enhance our understanding of the immediate effects of electrical stimulation when applied in different locations and using different waveforms, to help us determine the optimal parameters for functional recovery in people living with SCI.

We hypothesized that: (1) PAS (TMS paired with tSCS), which are timed to coincide at the spinal motoneurones will facilitate corticospinal excitability and motor output to a greater extent than PAS where the inputs do not coincide at the motoneurones; (2) short trains of tSCS will facilitate corticospinal excitability to a greater extent than short trains of PNS, and; (3) short trains of tSCS will facilitate corticospinal excitability to a greater extent than short trains of tSCS, which are modulated with a carrier frequency in the kHz range, when delivered at similar current intensities.

## Materials and Methods

This study was carried out at the Sobell Department of Motor Neuroscience and Movement Disorders at Queens Square and the Aspire CREATe laboratories at the Royal National Orthopaedic Hospital (RNOH). Ethical approval for the study was provided by UCL Research Ethics Committee (protocol IDs: 5732/002, 6864/001 and 6864/006), and all participants gave informed written consent prior to participating in the study. Inclusion criteria were: greater than 18 years old and no previous neurological or musculoskeletal problems relating to their back or lower limbs. Exclusion criteria were: history of epilepsy; implants or metal in the head (other than dental) or close to the electrode sites; previous neurosurgery. None of the participants had contraindications to TMS (Rossi et al., 2011). All experiments were carried out on healthy participants.

Three experiments were designed to evaluate the effects of tSCS on spinal and corticospinal excitability. Experiment I was designed to assess the short term effects of PAS (TMS paired with tSCS) on spinal and corticospinal excitability, and motor output. Experiment II compared the immediate effects of activating the same nerve but in different locations: at the spinal cord (tSCS) versus over the nerve more distally in the lower limb (PNS) on corticospinal excitability (amplitude of motor evoked potentials (MEPs)). Experiment III was used to directly compare the immediate effects of tSCS with tSCS modulated with a carrier frequency in the kHz range, on corticospinal excitability (MEP amplitude). This type of tSCS is being used increasingly in clinical trials (as it is thought to reduce discomfort) but there is currently limited evidence to support its usage over conventional waveforms.

### Experiment I: Paired Associative Stimulation

Twenty-four participants (13 males; 11 females) visited the laboratory on two occasions, separated by a week. Mean (SD) age was 23 (4) years. In each session a different PAS inter-stimulus interval (ISI) was investigated: PAS_0ms_, reflecting the approximately identical arrival of afferent and corticospinal volleys at the spinal motoneurones (i.e., zero-time delay); and PAS_5ms_, where the afferent volley arrived at the spinal motoneurones 5 ms before the corticospinal volley, taking into account conduction time as indicated in section ‘Conduction Time Measurements’. Participants were blinded to which PAS intervention they were receiving, and the order of interventions was randomized. Data collection and analyses were performed by separate investigators, with the analyses performed blind to the condition.

**FIGURE 1:**
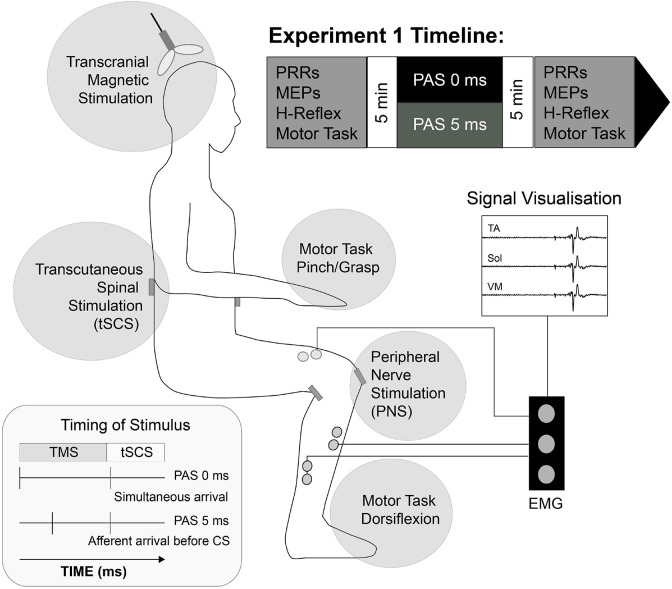
Schematic representation of Experiment I setup. Transcutaneous stimulating electrodes were placed paravertebral at landmarks over T12/L1 to L3/4 spinous processes (tSCS) and the popliteal fossa (PNS). Pairs of surface EMG electrodes were placed over tibialis anterior (TA), soleus (Sol) and Vastus Medialis (VM) muscle groups bilaterally and corticospinal (MEPs), spinal (PRR), peripheral (H-Reflex) activity and motor task response (Force) was evaluated 5 min before and after selected Paired Associative Stimulation (PAS) protocol. PAS lasted ∼14 min, and consisted of 100 pairs of transcranial magnetic stimuli applied to leg motor cortex and non-invasive, transcutaneous spinal cord stimuli to participants in the seated position.

During each session, participants sat in a chair, feet relaxed on the ground and knees/ankles angled at ∼90° (Figure 1). The participants right foot was positioned in a custom-built isometric dynamometer to allow measurement of dorsiflexion forces. The strain gauge was positioned securely over the dorsum of right foot (distal portion of the metatarsals). The positions of the chair and strain gauge were individualized and maintained for each person across sessions. Pairs of surface electromyography (EMG) electrodes (WhiteSensor 40713, Ambu^®^) were placed over the tibialis anterior (TA), soleus (SOL) and vastus medialis (VM) muscles of the participant’s lower limbs, each pair ∼3 cm apart and positioned according to SENIAM guidelines (Hermens et al., 2000). EMG data was amplified (x1000) and filtered (between 10 and 500 Hz) using a Digitimer Isolated Patient preamplifier/amplifier system (D360 8-channel Patient Amplifier System, Digitimer, Welwyn Garden City, Hertfordshire, United Kingdom) digitized at 5 kHz (Power 1401, Cambridge Electronic Design, Cambridge, United Kingdom), and sampled into data acquisition software (Signal v6.04, Cambridge Electronic Design, Cambridge, United Kingdom). The data were stored on a personal computer for offline analysis.

Transcranial Magnetic Stimulation (TMS) was applied with a Magstim 200^2^ (Magstim Co., Ltd., United Kingdom) and a 70 mm diameter double cone coil (Posterior-Anterior current direction). The coil was placed over the leg area of the primary motor cortex, just lateral and anterior to the vertex (Rossini et al., 2015), to activate the lower limb and determine the motor hotspot for TA (the area in which MEPs were largest and most consistent). Resting motor threshold (RMT) was determined individually for each participant, and defined as the lowest stimulation intensity that evoked peak-to-peak responses in the TA of >0.05 mV in at least 50% of trials in a series of 10 pulses (Rossini et al., 2015). A truncated input-output curve was generated by delivering stimuli at incremental intensities in steps of 5% stimulator output from 50 % until a plateau in MEP amplitude or 100% maximum stimulator output was reached, solely with the intention of determining the MEP_Max_ and doing so as efficiently as possible. A plateau was defined as when the MEP amplitude was broadly consistent across three consecutive stimulus intensities. Measurements were repeated for each leg/hemisphere.

Transcutaneous spinal cord stimulation was applied using a constant current stimulator (DS8R, Digitimer, United Kingdom) delivering monophasic square wave pulses 1ms in duration. It should be noted that monophasic pulses are not charge-balanced, which can result in damage at the electrode-tissue interface due to chemical reactions related to charge build-up when using pulse trains. A single 5 cm x 5 cm cathode (MedFit 1^®^ Extra TENS Electrode) was initially placed centrally over the L3/4 intervertebral space. The iliac crests were used as surface markings to find L4. The optimal site was found by delivering tSCS stimuli and monitoring PRRs in the TA: this was generally T12/L1 to L3/4 consistent with the literature (Roy et al., 2014; Gerasimenko et al., 2015a). Electrode position was kept consistent within individuals across sessions. Two 10x10cm carbon rubber anodes were placed bilaterally on either side of the umbilicus, on the iliac crests, just above the anterior superior iliac spines (Dixon et al., 2016). Stimulation intensity was first increased until it produced a clear motor response. We then attempted to confirm that this was a PRR, rather than a result of direct activation of motor axons. Two pulses at the same intensity were delivered 50 ms apart (Minassian et al., 2007). PRR was confirmed if the response to the second stimulus was suppressed, which is indicative of post-activation depression, reflecting a prolonged refractory period (Crone and Nielsen, 1989; Minassian et al., 2007; Sharpe and Jackson, 2014). A lack of depression indicated that motor, rather than sensory, axons were activated.

PRR threshold was determined as the lowest stimulation intensity (mA) at which tSCS evoked peak-to-peak responses of >0.05 mV in the TA in at least 50% of 10 consecutive stimuli (Rossini et al., 2015). An input-output curve was generated by increasing the stimulations in steps of 5mA, until a plateau in PRR amplitude was reached, in order to determine the PRR_Max_.

#### Conduction Time Measurements

Conduction time measurements of the descending motor and ascending sensory pathway segments were used to determine relative TMS/tSCS timings during the PAS and conditioning protocols, and calculated for each individual in each session (Figure 1). They were determined for the right TA muscles, because PAS was intended to target the right leg specifically. The following conduction times were calculated using standard methods (Rossini et al., 2015).

Peripheral motor conduction time (PMCT) is time taken for volleys generated at the motoneuron soma be transmitted to the muscle, calculated using the F-wave methodology. The F-wave latency includes a 1ms “turn-around time” at the soma (Rossini et al., 1987).

$$\text{PMCT}\,\left(m\,s\right)=\frac{\begin{array}{c} (F\mathrm{wave}\mathrm{latency}\left(\mathrm{ms}\right)+\\ M\mathrm{wave}\mathrm{latency}\left(\mathrm{ms}\right)-1ms) \end{array}}{2}$$

Central motor conduction time (CMCT): time taken for volleys generated in the cortex to travel from M1 to the spinal motoneurons (Rothwell et al., 1991).

CMCT⁢(m⁢s)=MEP⁢latency⁢(ms)-PMCT⁢(ms)

Afferent conduction time (ACT): time taken for tSCS evoked afferent volleys to travel to the spinal cord motoneuron.

ACT⁢(m⁢s)=PRR⁢latency⁢(ms)-PMCT⁢(ms)

MEP, F- wave-, M-wave and PRR latencies were all defined as the onset at which the EMG response deviated from baseline. All latencies were viewed in Signal v5 software (CED, Cambridge, UK) and assessed visually as the last peak/trough before signal deflected above the range of the baseline noise.

First, MEP latency was determined, and represents the conduction time of the fastest conducting axons from M1 to the muscle. This was achieved by delivering 5 TMS pulses at maximal MEP amplitude (MEP_Max_) while participants maintained weak dorsiflexion contraction of their right foot (∼10% of maximal voluntary contraction), and measured as the interval between the TMS pulse and MEP onset. The shortest latency was taken for further analysis. Next, F- and M-waves were elicited via ES to the peroneal nerve at rest. Twenty stimuli were delivered 1s apart at an intensity 30% greater than that required to elicit a maximal M-wave. From these recordings, the earliest onset of M- and F-waves were determined. The maximal M-wave amplitude was also recorded before and after PAS to account for peripheral changes in excitability at the neuromuscular junction. PRR latency was determined using maximal PRR amplitude(PRR_Max_) recordings, to identify the earliest onset of PRR.

#### Paired Associative Stimulation Intervention

The PAS protocol consisted of delivering 100 pairs of sub-threshold TMS (95%RMT) and supra-threshold tSCS (130% PRR threshold) stimuli. Each pair was delivered 8s apart, and the total duration of PAS was ∼14 min. Sub-threshold TMS pulses were chosen to minimize stimulus spread to the other hemisphere, and help ensure that descending corticospinal volleys were largely restricted to the right leg. Supra-threshold PRR stimuli were chosen to ensure that a large population of spinal motoneurones would receive sufficient afferent input and thus be susceptible to summation of afferent and descending volleys. Two inter-stimulus intervals were used (PAS_0ms_ and PAS_5ms_) one in each session.

#### Motor Tasks

To examine whether persistent changes in corticospinal transmission following PAS would influence motor output during voluntary muscle contractions, two different behavioural tasks were utilized. A subset of participants was assigned to one or other of the two tasks, as performing both may have caused muscle fatigue.

The first involved ballistic contractions of the dorsiflexor muscles. Participants (*n* = 11) first performed two maximum voluntary contractions (MVCs) of the right dorsiflexor muscles, each lasting 2-3 s and separated by 20 s rest (following a series of submaximal warm-up contractions at 25, 50, and 75% perceived maximum force). Participants received real-time feedback of the force on a computer monitor on each trial. The instantaneous peak force was measured for each contraction and higher value was taken as the MVC force. Participants then perform 10 ballistic isometric contractions, each 15 s apart, using methods documented elsewhere (Folland et al., 2014). Briefly, a cursor on the screen displayed the ‘target,’ which was set at 80%MVC force. Participants were asked to exceed this target as abruptly as possible upon receiving a visual cue, emphasizing that the contraction should be “fast and forceful.” The peak rate of force development (PRFD; 10 ms smoothing window) from each trial was displayed on the screen and participants were encouraged to improve PRFD on each trial to ensure rapid movements. Participants were given 5 ‘practice’ trials at the start for familiarization.

The second behavioural task employed a force-matching paradigm adapted from Taylor and Martin (2009). They used a form of PAS employing peripheral nerve stimulation to produce unilateral changes in excitability of the elbow flexors, and examined the effects on motor performance by asking participants to perform brief, bilateral and matched force pulses. The outcome was that force on the PAS-targeted side tended to over- or under-shoot that of the unaffected side depending on whether excitability was modulated up or down. Since we were unsure about the laterality of effects with our PAS protocol, given that spinal stimulus was certainly bilateral and TMS possibly too, we could not use the same approach. We therefore asked participants to match hand (pinch) and foot (dorsiflexion) forces, assuming that PAS would not affect hand physiology or motor performance.

Participants (*n* = 13) first performed three MVCs of the right dorsiflexor muscles, each separated by 30 s, and then repeated the procedure with hand muscles using a pinch grip between the thumb and forefinger. Pinch force was measured using a strain gauge (Pinchmeter, Biometrics Ltd., United Kingdom). The signal was amplified (DataLog, Biometrics Ltd., United Kingdom) and digitized at 500 Hz (Signal v6.04, Cambridge Electronic Design, Cambridge, United Kingdom). Peak force was determined for each contraction and the greatest peak force across all contractions was taken as the maximum MVC force. Participants then practiced performing brief voluntary contractions to a given target (10% maximum MVC force), first with the dorsiflexors, then with the hand, and then with simultaneous dorsiflexor and hand contractions. They performed 40 trials of each because pilot data had shown that at least 20 trials were needed for the cumulative mean and standard deviation of the peak force to stabilize.

Each trial began with an auditory tone (500 Hz) cueing the participants to contract. Participants were encouraged to make a brief contraction to reach the target force within 1 s of the cue, which was delivered every 5 s, and not to attempt to make corrections if they felt they had over- or under-shot the force. These weak, brief contractions were employed to minimize fatigue and avoid feedback-related corrections in force. Visual feedback of the peak force relative to the target force was displayed after each trial for the separate pinch and dorsiflexor contractions. But for the combined pinch and dorsiflexor contractions, feedback was only given for the pinch, though they were not explicitly told whether the feedback was from the hand or foot. This meant that participants could not use feedback to adjust dorsiflexion force. After practice, participants performed a single block of 40 trials of the combined pinch and dorsiflexor contraction, 5 s apart, both before and after the PAS protocol. Absolute peak force (PF) was measured for each contraction, and the relative force of the dorsiflexor contraction with respect to the pinch force was expressed as a ratio.

#### Outcome Measures

Outcome measures were completed prior to and post the PAS intervention. Previous work using PNS has shown that the after-effects of a broadly similar PAS protocol emerge ∼4 min after the protocol (Taylor and Martin, 2009), therefore we waited 5 min before starting post-PAS measurements.

Changes in corticospinal excitability were assessed in all participants by delivering 20 TMS pulses at 0.2Hz at 130% RMT. Changes in afferent-motoneuronal excitability were assessed either by PRRs (*n* = 11), or H-Reflexes in the right SOL (*n* = 13). For PRRs, 20 tSCS pulses were delivered at 130% PRR threshold. For H-Reflexes, electrical stimulation was delivered to the tibial nerve (square-wave pulse, 1ms duration; DS8R, Digitimer, United Kingdom) with the cathode on the popliteal fossa and the anode ∼2 cm more proximal to the crease of the knee. H-reflex threshold was initially determined as the minimum intensity capable of producing a visible EMG response >0.05 mV. Before and after the PAS intervention, 20 stimuli were delivered at 120% H-reflex threshold; each delivered 8 s apart.

### Experiment II: Transcutaneous Spinal Cord Stimulation and Peripheral Nerve Stimulation

Twenty participants (12 male; 8 female) were recruited and attended one session in the lab. Mean (SD) age, height and weight were 28 (11) years, 172.8 (9.2) cm and 65.5 (11.5) kg. Participants were requested to lie supine on a medical examination couch for the duration of the experiment. Pairs of surface electromyography (EMG) electrodes (WhiteSensor 40713, Ambu^®^) were placed over the tibialis anterior (TA) and soleus (SOL) muscles of the participant’s right lower limb, as described in Experiment I (Section “Experiment I: PAS”).

During the experiment, participants received single pulses and short trains of either thoraco-lumber tSCS or peripheral nerve stimulation (PNS) targeting the TA muscle. Both were immediately followed by a single pulse of TMS. tSCS was set up as described in Experiment I (Section “Experiment I: PAS”), and was applied at 110% PRR threshold throughout the experiment. PNS was delivered to the common peroneal nerve (square-wave pulse, 1 ms duration; DS7a, Digitimer, United Kingdom) with the cathode positioned lateral and just inferior to the tibial plateau and the anode on the anterior aspect of the lower limb, inferior to the patella. Motor threshold was determined as the minimum intensity capable of producing a visible EMG response >0.05 mV in the TA muscle. PNS of the TA muscle was applied at 110% motor threshold throughout the experiment. TMS was set up as described in Experiment I (Section “Experiment I: PAS”) and delivered at 130% RMT throughout the experiment.

**FIGURE 2:**
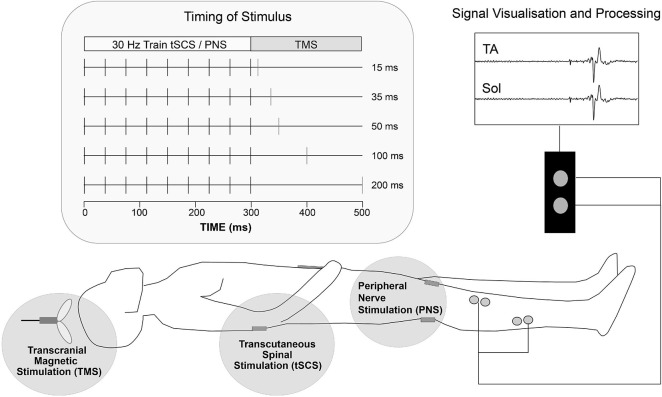
Schematic representation of experiments 2&3 setup. Transcutaneous stimulating electrodes were placed paravertebral at landmarks over T12/L1 to L3/4 spinous processes (tSCS) and the popliteal fossa (PNS). TMS was delivered over the motor cortex region for lower limb at various interstimulus intervals following tSCS or PNS to participants in the supine position. Pairs of surface EMG electrodes were placed over tibialis anterior (TA) and soleus (Sol) muscle groups and MEP activity was recorded.

Three testing blocks were carried out, separated by 15min rest periods; each block lasted approximately 10-15min and was made up of 86 trials, administered in a random order. Each trial consisted of a single pulse of tSCS (tSCS), or a 30Hz train (10 pulses) of tSCS (tSCST) or PNS (PNST), immediately followed by a single pulse of TMS, administered with an ISI ranging from 10 to 200 ms. Specifically, the ISIs tested were 10, 15, 20, 30, 40, 50, 75, 100, and 200 ms for tSCS, and 15, 30, 50, 100, and 200 ms for tSCST and PNST (Figure 2). Each test block consisted of 10 TMS pulses alone (control) and 4 x each ISI for tSCS (36 trials), tSCST (20 trials) and PNST (20 trials). Therefore 86 trials were administered in total within each block, with 7 s rest provided between each trial. This provided a total of 30 control trials and 12 trials at each ISI for each of the three test conditions (tSCS, tSCST, and PNST).

### Experiment III: Transcutaneous Spinal Cord Stimulation and HF-Transcutaneous Spinal Cord Stimulation

Ten participants (4 male; 6 female) were recruited and attended one session in the lab. Mean (SD) age, height and weight were 32 (9) years, 172.8 (10.3) cm and 66.4 (11.4) kg. Participants were requested to lie supine on a medical examination couch for the duration of the experiment. Pairs of surface electromyography (EMG) electrodes (WhiteSensor 40713, Ambu^®^) were placed over the tibialis anterior (TA) and soleus (SOL) muscles of the participant’s right lower limb, as described in Experiment I (Section “Experiment I: PAS”).

During the experiment, participants received either conventional tSCS (monophasic pulses with a 1ms pulse width) or tSCS incorporating frequency modulation with a carrier frequency of 9090Hz (HF-Burst tSCS): a single HF-burst of tSCS contained 10 monophasic pulses, each with a 50μs pulse width, with an overall burst duration of 1ms. tSCS was set up and threshold intensity was identified as described in Experiment I (Section “Experiment I: PAS”), for both tSCS and HF-burst tSCS (tSCS_B_) waveforms.

Four tSCS conditions were then assessed; (i) tSCS, (ii) tSCS_B_, (iii) tSCST, and (iv) 30Hz train (10 bursts) of HF-burst tSCS (tSCST_B_). For all conditions, stimulation was applied at the motor threshold determined with tSCS. Motor threshold was additionally determined for tSCS_B_, however stimulation needed to be applied at a relatively high current (150-200 mA), and the subjects reported discomfort with these intensities, so all further experiments were performed at motor threshold determined for tSCS only. This is closer to what is done in the application of HF-tSCS in clinical trials: there is no clear consensus on how HF-tSCS intensity is defined, some determine it using PRR threshold from single tSCS pulses (Benavides et al., 2020), as we did, and others determine it based on feedback from the participant (Rath et al., 2018; Sayenko et al., 2019). Intensities between 30 and 120 mA are typically applied. Each condition was repeated 5 times, in a random order. To assess the interaction of tSCS with descending drive, two test blocks were completed, one using each of the two waveforms (tSCS, and tSCS_B_), in a random order. Each block consisted of 50 trials: 20 single pulses or single bursts of tSCS, 20 pulse trains or burst trains of 30Hz tSCS immediately followed by a single pulse of TMS administered at an ISI of 50 ms and 10 single pulses of TMS alone.

### Data Analysis

All data are presented as mean ± standard deviation (SD), unless stated otherwise. All statistical tests were conducted using IBM SPSS Statistics for Windows, Version 27.0. Normal distribution was tested by the Shapiro-Wilk’s test; where data was not normally distributed, log or square root transformations were performed and normality rested.

#### Experiment I: Paired Associative Stimulation

Peak-peak MEP and PRR amplitudes were measured from TA, SOL and VM EMG traces, and averaged across all pre- and post- stimulation trials, respectively. Peak-peak H-Reflex amplitudes were measured from SOL EMG traces, and averaged across all pre- and post- intervention trials. From the maximal ballistic contractions, PF and PRFD were measured. The root mean squared EMG activity for TA and SOL (agonist/antagonist) during each contraction over the first 50 ms from EMG onset were also measured as a marker of neural drive, as these may explain any changes in PRFD (Folland et al., 2014). For the force matching task, absolute PF and the ratio of dorsiflexion to pinch force were measured for each contraction, and averaged across all trials.

Paired t-tests were used to determine differences between PAS intervals [PAS_0ms_, PAS_5ms_] for H-reflex amplitude, PF and PRFD during the ballistic contractions and PF and force ratio during the force matching task. Analysis of MEPs, PRRs, and changes in EMG during the ballistic contractions, began with a two-way ANOVA to observe main effects of PAS interval (Session [PAS_0ms_, PAS_5ms_] between muscles [TA, SOL, VM]) and paired t-tests were performed as *post hoc* comparisons to determine the specific nature of any differences identified in the ANOVAs. Extreme outliers were identified using z-scoring and subsequently excluded from the statistical analysis.

#### Experiment II: Transcutaneous Spinal Cord Stimulation and Peripheral Nerve Stimulation

Peak-peak MEP amplitude was measured for each trial from TA and SOL EMG traces. For each block, per-participant data for each ISI were averaged within each condition. For each ISI, data were compared between the three testing blocks using one-way ANOVAs. Data from the three blocks were then averaged for each participant. To assess changes in MEP amplitude over the range of ISI’s used for tSCS, tSCST, and PNST, one-way ANOVAs were used. MEP amplitude during tSCST and PNST, across the range of ISI’s used, were compared using two-way repeated measures ANOVA (stimulation type x ISI). Where significant effects were identified, *post-hoc* analysis was carried out using paired t-tests, with a Bonferroni correction for multiple comparisons.

#### Experiment III: Transcutaneous Spinal Cord Stimulation and HF-Transcutaneous Spinal Cord Stimulation

Peak-peak MEP amplitude was measured for each trial from TA and SOL EMG traces. For each block, per-participant data for each waveform were averaged within each condition (pulse/train). Averages were normalised to control (TMS-alone MEP Peak-to-peak). Data was compared using Two-way ANOVA with ‘Waveform and ‘Condition’ as the two factors. If the ANOVA revealed a significant interaction between factors, simple main effects were assessed. Where significant main effects were identified, *post-hoc* analysis was carried out with a Bonferroni correction for multiple comparisons.

## Results

### Experiment I: PAS

**FIGURE 3:**
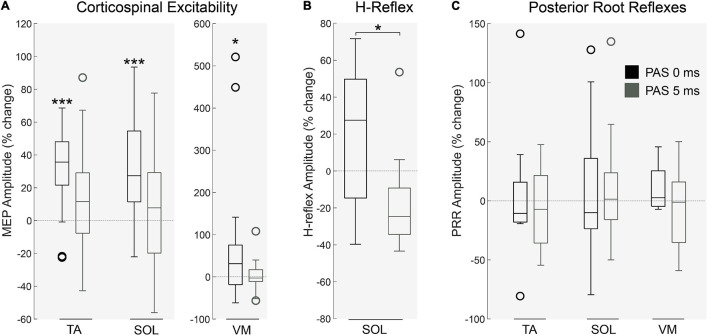
Physiological changes following PAS protocol. **(A)** Corticospinal excitability in the right lower leg muscles was significantly altered by PAS interval (*p* < 0.01, *n* = 22) in the TA (*p* < 0.001), SOL (*p* < 0.001) and VM (*p* < 0.05) following PAS_0ms_. This effect was similarly identified in the **(B)** amplitude of H-reflexes (in the SOL muscle), significantly increased by PAS_0ms_ (*n* = 13, *p* < 0.05). Interestingly, **(C)** spinal root reflexes in the lower limb were unaffected by both PAS protocols (*n* = 10, *p* > 0.05). Each box indicates the Upper (25 percentile) and Lower (75 th percentiles), the whiskers represent the minimum and maximum values, the central line is median value and open circles represent outliers (values that are more than 1.5 of the interquartile range away from the top or bottom of the box). *p < 0.05 ***p < 0.001.

A Two-way ANOVA revealed a significant main effect of PAS interval on corticospinal excitability (*p* < 0.01; Figure 3; MEP amplitude; *n* = 22). Following PAS_0ms_ a significant increase in MEP amplitude in the TA (*p* < 0.001), SOL (*p* < 0.001) and VM (*p* < 0.05) was found. This coincided with an increase in H-reflex amplitude in the SOL muscle following PAS_0ms_ (*n* = 13, *p* < 0.05, Figure 3). As predicted, corticospinal excitability was unaffected following PAS_5ms_ (Figure 3). PRRs were unaffected by both PAS protocols (*p* > 0.05; PRR amplitude; *n* = 10; Figure 3).

**FIGURE 4:**
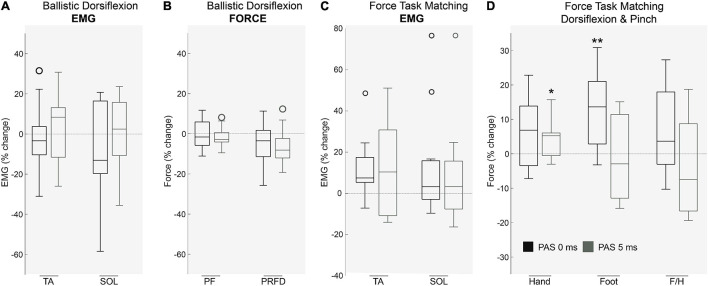
The effects of PAS on voluntary motor tasks. During ballistic dorsiflexion both muscle activation of **(A)** agonist/antagonist muscles and force including **(B)** Peak Force (PF) and Peak Rate of Force Development (PRFD) were unaffected by PAS protocols (*p* > 0.05, *n* = 11). During a force-task matching exercise (pinch and dorsiflexion) Muscle EMG **(C)** remained unaffected (*p* > 0.05, *n* = 11), however, **(D)** Force production was significantly affected (*p* < 0.01, *n* = 11) in the hand after PAS_5__Ms_ (*p* < 0.05) and in the foot after PAS_0__Ms_ (*p* < 0.01). Each box indicates the Upper (25 percentile) and Lower (75 th percentiles), the whiskers represent the minimum and maximum values, the central line is median value and open circles represent outliers (values that are more than 1.5 of the interquartile range away from the top or bottom of the box). *P < 0.05 **P < 0.01.

We observed no change in lower limb EMG (*p* > 0.05; Figure 4A; *n* = 10) and force production (*p* > 0.05; Figure 4B; *n* = 11) during ballistic dorsiflexion contractions following either PAS protocol. Dorsiflexion EMG in a force matching task was unaffected by PAS interval (*p* > 0.05; *n* = 11), however, dorsiflexion force increased after PAS_0ms_ (*n* = 11; *p* < 0.01), and Foot/Hand ratio tended to increase (although this did not reach statistical significance (*n* = 11; *p* = 0.11)), hinting that foot force increased relative to hand force for PAS_0ms_ but not PAS_5ms_.

### Experiment II: Transcutaneous Spinal Cord Stimulation and Peripheral Nerve Stimulation

**FIGURE 5:**
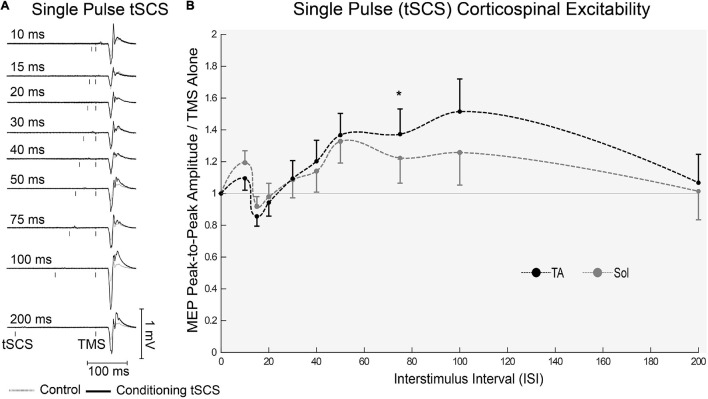
Effects of single pulses of tSCS delivered at various ISIs on TMS evoked potentials in the lower limb. **(A)** Example waveform average (10-control, 4-conditioned trials) EMG trace responses evoked in the TA from one participant under control (gray dashed line) and tSCS conditioned (solid black line) at each ISIs. **(B)** Normalized peak-to-peak MEP amplitude in the TA (black dashed line) and Sol (gray dashed line) muscles at interstimulus intervals. Mean ± SEM; *P < 0.05.

**FIGURE 6:**
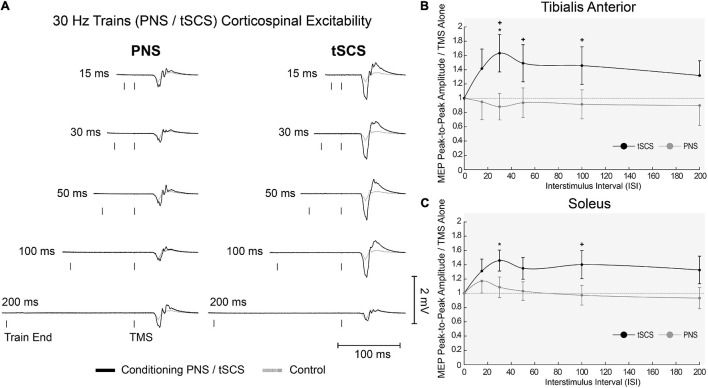
Effects of trains of tSCS (30 Hz) delivered at various ISIs on TMS evoked potentials in the lower limb. **(A)** Example waveform average (10-control, 4-conditioned trials) EMG responses evoked in the TA from one participant under control (gray dashed line) and tSCS/PNS conditioned (solid black line) at each ISIs. **(B)** Normalized peak-to-peak MEP amplitudes following either tSCS (black solid line) or PNS (gray solid line) in the TA and **(C)** Sol muscles at interstimulus intervals. Mean ± SEM; *P < 0.05.

One participant withdrew from the study after completing the first block, due to discomfort of the tSCS. All other participants completed the full experiment and data analysis was carried out on *n* = 19. Representative EMG responses to single pulses of tSCS are shown in Figure 5A. Following tSCS, the change in MEP amplitude showed a similar pattern in TA and Soleus muscles (Figure 5B). At short ISI’s < 30 ms both muscles were slightly inhibited, and then tended towards facilitation from 30 to 100 ms. This facilitation was significant at an ISI of 75 ms for the TA muscle only (*p* < 0.001). For tSCST, MEP amplitude tended to be facilitated for both TA (Figure 6A) and Soleus muscles, which was significant at an ISI of 30 ms for both muscles (*p* < 0.01). No change in MEP amplitude was observed for PNST in both muscles (*p* > 0.05). In comparison to PNST, MEP amplitude following tSCST was significantly higher at ISIs of 30, 50, and 100ms for the TA, and 100ms for the soleus muscle (Figures 6B,C).

### Experiment III: Transcutaneous Spinal Cord Stimulation and HF-Burst Transcutaneous Spinal Cord Stimulation

Mean (SD) motor thresholds determined for tSCS (*n* = 10) and tSCS_B_ (*n* = 6) were 46.8 (7.8) and 180.7 (14.2) mA, respectively. tSCS_B_ required significantly higher currents to elicit lower limb posterior root-muscle reflexes compared with tSCS. We limited stimulation current to a maximum of 200 mA or maximum tolerable by the participant (whichever was lower). Using HF-burst tSCS, these limits were reached before reflexes were obtained in some participants.

**FIGURE 7:**
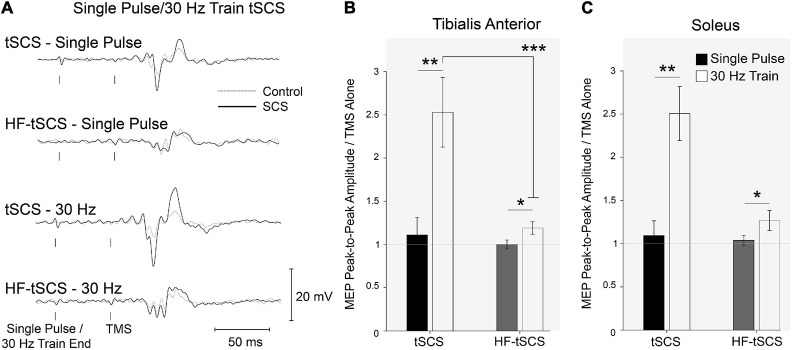
The effects of two separate waveforms (Traditional tSCS and High-frequency tSCS). **(A)** Example waveform average (20-control, 10-conditioned trials) EMG responses evoked in the TA from single participant under control (gray dashed line) and tSCS/HF-tSCS conditioned (solid black line) with single pulses or 30 Hz trains. Normalized peak-to-peak MEP amplitudes in tSCS and HF-tSCS waveform conditions in the **(B)** TA and **(C)** Sol muscles at interstimulus intervals. Mean ± SEM; *P < 0.05; **P < 0.01; ***P < 0.001.

Representative EMG responses to all conditions are shown in Figure 7A. MEP amplitude was unaltered when conditioned by single pulses or single bursts across both waveforms (tSCS and tSCS_B_). A Two-way ANOVA revealed significant effects for each condition (*p* < 0.01, *n* = 10) and a significant interaction (*p* < 0.001) in the SOL whereas the TA displayed significant effects for both waveform (*p* < 0.01) and condition (*p* < 0.001) with a similar level of interaction (*p* < 0.001). Pre-conditioning with trains of tSCS (ISI 50ms) significantly increased peak-to-peak MEP amplitude for both waveforms (tSCST (*p* < 0.01) and tSCST_B_ (*p* < 0.05)) for both SOL and TA muscles (Figures 7B,C), *n* = 10). Facilitation was significantly greater following trains of tSCST than tSCST_B_ in the TA (*p* < 0.001; Figure 7B, *n* = 10).

## Discussion

The present studies have explored, in healthy humans, two potential mechanisms by which tSCS may enhance functional recovery: (i) the effects of repeated pairing of descending and afferent volleys at the spinal cord, and; (ii) the immediate effects of afferent input on corticospinal excitability. In the first experiment, we found that repeated and coincidental input to motoneurones from descending corticospinal and ascending afferent input produced a short-term (lasting at least 5 min) increase in corticospinal excitability, which influenced motor performance. In the second experiment, we showed that short trains of tSCS applied at motor threshold immediately facilitated subsequent motor-evoked potentials, at inter-stimulus intervals of 30-50 ms. This facilitation was not observed following trains of peripheral nerve stimulation, however it was present when using ultra-HF burst (10 kHz) tSCS waveforms, applied at sub-motor threshold intensities, although to a much smaller extent.

### Experiment I: Lasting Effects of Paired Associative Stimulation on Corticospinal Physiology and Motor Performance

Physiologically, we expected a lasting facilitatory effect of repeated pairs of descending (TMS) and afferent spinal (tSCS) volleys, particularly when they were timed to arrive coincidentally (PAS_0ms_) (Roy et al., 2014; Mishra et al., 2017) because the repeated summation of volleys should produce repeated paired pre- and post-synaptic potentials at the α-motoneuron (Bi and Poo, 1998; Taylor and Martin, 2009). Indeed, we observed a lasting facilitatory effect in the lower limb following PAS_0ms_ in both corticospinal MEPs and H-Reflexes. PAS_5ms_ did not elicit facilitation, indicating that PAS effects are sensitive to the interval between the arrival of afferent and descending volleys. The corticospinal facilitation following PAS_0ms_ was likely due to an increase in spinal excitability, as has been found in rodents (Mishra et al., 2017). Dixon et al. (2016) explored a similar tSCS-TMS PAS protocol in healthy subjects, and reported a reduction in H-Reflex mediated post activation depression following the intervention, but also found a reduction in corticospinal excitability, which disagrees with our findings. In their study, corticospinal excitability was assessed based on TA MEP recruitment curves, and the reduction was only observed at 1.3 times of 50 % maximal MEP stimulation before PAS. MEP amplitudes in our study were measured at 130% of resting motor threshold, and recruitment curves were not repeated after the intervention, therefore it is difficult to directly compare these findings. We speculate that the effects were due to a form of STDP, though future studies might want to consider examining PAS effects across a broader range of intervals to test for the presence of the classic long-term potentiation/long-term depression effects resulting from variations in the interval between inputs (Bi and Poo, 1998). We observed no change in PRR excitability following either PAS protocol. Previous studies have shown a time-dependent increase in H-reflex amplitude with combined peripheral and cortical stimulation with selectivity of nerve stimulation (Poon et al., 2008; Cortes et al., 2011). Stimulation of posterior roots is known to be much less specific and can cause motor responses in multiple muscles bilaterally (Minassian et al., 2007). Therefore, the changes in Ia-motoneuronal transmission or motoneuronal excitability may have been obscured by complicated interactions (e.g., reciprocal inhibition, crossed effects) when we measured PRRs. Indeed, recovery of the PRR has been reported to be substantially supressed compared to recovery of H-Reflexes, in neurologically intact subjects (Hofstoetter et al., 2019).

We predicted that if PAS_0ms_ effectively increased corticospinal excitability, it would facilitate ballistic motor performance by strengthening synaptic connections between corticospinal drive and spinal motoneurons (Bunday and Perez, 2012). Our results indicate that PAS_0ms_ did not alter muscle activity or force output during ballistic dorsiflexion. Ballistic contractions are more suitable for examining motor performance than contractions focusing simply on maximal force, because whilst most individuals can voluntarily attain ∼90 % of a muscle group’s capacity for maximal force production (Folland and Williams, 2007), they typically only achieve ∼40% of its capacity for ballistic force production (measured as the rate of force development (Folland et al., 2014). Thus there is more scope for improving ballistic force production. However, maximal ballistic contractions were still not improved following either PAS treatment, although this does not preclude the possibility of improving maximal force in people with weak corticospinal input, such as following SCI.

In contrast to the ballistic contractions, we found that dorsiflexion force after PAS_0ms_ was increased when performing sub-maximal contractions in a hand-foot force matching task. This task was adapted from Taylor and Martin (2009), who found it was sensitive enough to detect changes in motor output in the targeted side compared to the contralateral side, depending on modulations in excitability due to the PAS intervention. As our intervention could not target one side specifically, we compared motor output from the lower limb to the upper limb, which was not targeted by our PAS intervention. We found that foot force was increased relative to hand force, demonstrating the specificity of the effects and hinting that PAS_0ms_ may have the effect of facilitating weak corticospinal input. We note that the effects were rather small, but this is perhaps not surprising given the brief duration of the PAS protocol and the fact that it was only a single session. In any case, the results are consistent with the idea that STDP-like processes that conceivably occur when tSCS is delivered during volitional movements as part of therapy could partly underlie the therapeutic benefits of combined tSCS. Furthermore, we speculate that effectiveness of rehabilitative interventions following SCI may be enhanced by providing a session of PAS_0ms_ prior to training sessions as a means to prime the CNS.

An important consideration for the PAS protocol is the extent to which the estimated conduction times were accurate, since this influences the degree of coincidence between the descending and afferent volleys recruited by TMS and SCS, respectively. The methods used to estimate CMCT and PMCT were based on standard procedures used in clinical neurophysiology (Rossini et al., 2015). In order to achieve the planned 0 and 5 ms difference in the arrival of descending and afferent volleys, it was necessary to estimate the approximate time at which the afferent volleys would arrive at the spinal motoneuron (ACT). We estimated the ACT using the same logic as for CMCT and PMCT, apportioning different parts of the total conduction time latency to conduction in different pathway segments (i.e. afferent and efferent nerves). In other words, we subtracted out the PMCT from the SCS reflex response, and assumed that the remainder reflected the conduction/transmission in the afferent nerves. Dixon et al. (2016) used a simpler and less individualized method of estimating CMCT, wherein CMCT is estimated as the MEP latency minus the SCS reflex latency plus a fixed 1.5 ms for afferent conduction and transmission time. Both methods gave approximately similar answers for CMCT (∼10.8 ms) and the afferent conduction/transmission time (∼1.5 ms). Hence, we assume the methods and outcomes are largely comparable.

### Experiment II: The Immediate Effects of Afferent Input (Transcutaneous Spinal Cord Stimulation or Peripheral Nerve Stimulation) on Corticospinal Excitability

Single pulses of tSCS caused facilitation of cortically evoked potentials, which was evident in the TA muscle at ISIs of 50-100 ms (although statistical significance was only achieved at the 75 ms ISI). In agreement, previous studies have found that tSCS facilitated cortically evoked potentials in the TA muscle at latencies of 50 ms (Knikou, 2014) or 100-150 ms (Roy et al., 2014). Previously, several studies have shown that afferent input from the muscle (single pulse PNS) facilitates cortically evoked potentials in muscles innervated by the stimulated nerve and in nearby muscles at ISIs of ∼50 ms (Nielsen et al., 1997; Petersen et al., 1998; Rosenkranz and Rothwell, 2003; Roy and Gorassini, 2008). This facilitation was thought to be cortical in origin, as it was associated with depression of intracortical inhibitory circuits and facilitation of intracortical excitatory circuits (Roy and Gorassini, 2008). Similar intracortical mechanisms could potentially explain the facilitation seen here after tSCS. Effects of single pulse tSCS on soleus MEPs in our study were less consistent: MEP facilitation was evident, following a similar trend as TA, but did not attain statistical significance. One previous study reported no facilitation in the soleus MEP following tSCS at ISI’s up to 200ms (Roy et al., 2014), and another reported significant facilitation at 50ms (Knikou, 2014).

When tSCS was applied in short 10-pulse trains (30 Hz), cortically evoked MEPs were facilitated for both the TA and soleus muscles at ISIs of 30-100 ms. The effects were more prominent with TA, which is likely due to its stronger innervation by the corticospinal tract. Although we expected trains of PNS (applied at 30 Hz) over the tibial nerve to have similar facilitatory effects, we found it had had no significant effect on MEP amplitude in the soleus muscle or the heteronymous TA muscle at ISIs of 15-200 ms. The reason for the lack of facilitatory effects is unclear, because stimuli delivered in trains with pulses >50 ms apart should avoid the refractory period of motoneurons and allow temporal summation of EPSPs across successive pulses. It is possible that short-term post activation depression caused by prior stimulation pulses within the PNS train supressed any facilitatory effects seen after a single pulse of PNS (Burke et al., 2001; Dean et al., 2014). Alternatively, it is possible that the pulse train was too short. PNS at 30 Hz has been shown to recruit more motor units the longer the stimulation train is applied (Dean et al., 2014), therefore trains longer than 10 pulses may have facilitated subsequent MEPs.

The PNS condition intended to activate one or two mixed peripheral nerves as is typical with PNS; in contrast, due to the organization of the spinal anatomy under the stimulating electrodes, tSCS is likely to simultaneously recruit several posterior roots, bilaterally (Minassian et al., 2007; Sayenko et al., 2015; Calvert et al., 2019). This will result in activation of multiple sensory afferents of which have heteronymous effects on motoneuron pools causing not only an increased amount of pre-synaptic inhibition but also homosynaptic depression (Maertens de Noordhout et al., 1988; Hofstoetter et al., 2019). The spread of activation may also have influence over multiple intra-spinal circuits, as well as supraspinal effects. Based on our data, we are unable to speculate on whether this facilitation is cortical in origin. However, recent findings suggest that facilitation of MEPs following 20 min of tSCS is cortical in origin and, interestingly, changing the stimulation waveform (to the 10kHz HF bursts) can alter this effect (Benavides et al., 2020). Tonic SCS has also been hypothesized to influence voluntary drive to motoneurones following SCI pre-clinically (Ichiyama et al., 2008; Van den Brand et al., 2012) and clinically (Harkema et al., 2011; Angeli et al., 2014).

### Experiment III: Spinal Cord Stimulation and Sub-Threshold HF-Burst Spinal Cord Stimulation Had Facilitatory Effects on Motor Evoked Potentials

Given that tSCS has typically been applied continuously at 15-30 Hz, either in traditional biphasic waveforms (Hofstoetter et al., 2013, 2015; Al’joboori et al., 2020) or in HF (10kHz) bursts (Gerasimenko et al., 2015c; Gad et al., 2017; Sayenko et al., 2019), we compared their immediate effects on corticospinal excitability. HF waveforms minimize the discomfort of traditional waveforms applied transcutaneously at similar currents. We found that the current required to elicit motor responses (PRRs) in the lower limb with single burst of HF tSCS (tSCS_B_) was three times that of a single pulse of tSCS (181mA *versus* 47mA, respectively), demonstrating that a much higher current is required to activate the posterior roots when incorporating frequency modulation with a carrier frequency in the kHz range, presumably due to the reduced injected charge. While the overall width of each waveform is the same (1 ms pulse width for tSCS and 1 ms burst duration for a single HF-burst), the actual amount of charge delivered during HF-burst tSCS is half that delivered during tSCS (Miller et al., 2016): one HF-burst of tSCS contains 10 monophasic pulses, each with a 50 μs pulse width. When tSCS_B_ was delivered at motor threshold (∼180 mA), participants reported discomfort similar to or greater than that experienced when tSCS was delivered at motor threshold (∼45 mA). In agreement, a recent study reported significantly higher current intensities to attain PRR threshold when tSCS was modulated with a 10kHz carrier frequency compared with conventional biphasic pulses (195 mA vs. 70 mA), and that discomfort between the two paradigms was similar when stimulation intensity was normalized to PRR threshold (Manson et al., 2020). Due to the substantially higher currents required to reach motor threshold, and the resulting discomfort, we explored the effects of tSCS_B_, delivered at a sub-threshold current intensity (i.e., at tSCS motor threshold, ∼45mA), on CST excitability.

Our data showed that 10-burst trains of HF tSCS (delivered at ∼25% motor threshold) facilitates descending motor drive to the lower limb; however, 10-pulse tSCS trains (traditional square waveforms delivered at motor threshold) had a significantly greater facilitatory effect. Presumably the facilitatory effect of HF tSCS would have increased if it were delivered closer to motor threshold, but this would not have the benefit of reduced discomfort. It would be interesting to explore whether sub-threshold trains of tSCS have similar facilitatory effects as sub-threshold trains of HF tSCS. Much less is known about the underlying mechanisms of HF-burst tSCS than tSCS, but they are thought to be very different (Linderoth and Foreman, 2017). SCS has been used widely in the treatment of chronic pain (neuropathic or ischaemic in origin), where the sensation of paraesthesia is used to set the stimulation intensity (Chakravarthy et al., 2018). More recently, HF-burst SCS has also been shown to be beneficial for pain management in individuals with chronic back and lower limb pain (Kumar et al., 2008; Kapural et al., 2015, 2016), in the absence of paraesthesia (Miller et al., 2016). Interestingly, SCS has been shown to reduce pain in rats at sub-paraesthesia thresholds (Koyama et al., 2018). Some mechanisms thought to account for this pain inhibition include increased transmitter release (serotonin and noradrenaline), inhibition of ascending sensory information and spinal dorsal horn interneuronal inhibition (Foreman and Linderoth, 2012). In particular, Aβ- large diameter non-nociceptive fibers have been associated with SCS induced paraesthesia due to their size and location in the dorsal columns (Holsheimer et al., 2011). Stimulation of Aβ-fibers can cause both activation of second order neurons and interneuronal inhibition in the dorsal columns. This indicates that SCS has the potential to alter ascending signals, and therefore modulate intra-cortical inhibition via somatosensory and primary motor cortex interactions (Turco et al., 2018). Recent findings suggest that HF-burst tSCS has an enhanced suppressive effect on cortical CST excitability compared to tSCS (Benavides et al., 2020). In their study, current intensity was the same for HF-tSCS and tSCS interventions, determined based on PRR threshold from a single biphasic pulse, similar to the protocol used in our study. Following 20 min of HF-burst tSCS, they observed a facilitation in sub-cortical but not cortical excitability in both SCI and healthy volunteers (Benavides et al., 2020). This coincided with an increase in intra-cortical inhibition. As this inhibition was only present following HF-burst tSCS and not tSCS, this suggests that different waveforms may act though separate mechanisms. Further investigation of frequency specific, pulse width and current amplitude properties at specific stimulation sites are required to estimate potential dose-response characteristic of tSCS and identify associated mechanisms.

## Clinical Implications

Transcutaneous spinal cord stimulation is currently being trialed for the purposes of aiding autonomic and functional recovery after SCI. There has been encouraging results from early clinical trials, however, the mechanisms underlying the effects of tSCS remain unknown. We have shown that single pulses of tSCS can immediately enhance corticospinal excitability, and that simultaneous arrival of single pulses of tSCS and TMS at the alpha motoneurone is required to increase corticospinal excitability in the short-term (at least 5 min) after the intervention. As short trains of tSCS immediately enhanced corticospinal excitability to a much greater extent than single pulses, it follows that pairing short trains of tSCS with TMS may further enhance the observed effects of the PAS protocol. However, this intervention may prove impractical due to activation the back musculature with tSCS trains (given at or above motor threshold), causing discomfort and a change in the participant’s posture. The ability of tSCS, delivered in ultra-high frequency bursts (up to 10 kHz), to remove discomfort associated with tSCS, whilst still enhancing corticospinal excitability, remains an attractive characteristic. Our data, and that of others (Manson et al., 2020), suggests that this may also be achievable with the use of sub-threshold tSCS, eliminating the need for high frequency waveforms, but this requires further investigation. Trains of sub-threshold tSCS or HF tSCS paired with TMS may then be worth exploring. The combination of descending input from voluntary drive paired with tSCS trains should also be explored.

## Experimental Limitations and Future Work

It should be noted that the mechanisms observed here in healthy participants may not fully translate to people with SCI. Indeed, the corticospinal tract is often extremely disrupted following an SCI, whereas the reticulospinal tract typically shows more sparing, and is thought to be important in mediating recovery.

In Experiment I (PAS) only two ISIs were tested, therefore we cannot be certain that the effects were due to STDP. As we only showed an effect at one interval and not another, our results do indicate that PAS effects are sensitive to the ISI/relative time of volleys, and future studies might want to consider a range of positive and negative intervals between the stimuli to show positive/negative effects. In addition, based on our results, we cannot determine the duration of after-effects. Greater duration and intensity of stimulation have been shown to extend after-effects for inducing plasticity (Mishra et al., 2017); the precise influence of stimulus timings and the duration of after-effects remain to be established.

In experiment III, the comparison between tSCS and HF tSCS was done at only one ISI; future studies should explore the timecourse of these effects with HF tSCS. In addition, tSCS and HF tSCS at a similar intensity relative to motor threshold (i.e., tSCS at sub-threshold intensity) should be compared to better understand the different mechanisms with these different forms of stimulation. Another limitation was that we were not able to show latencies of ascending signals following tSCS or PNS and therefore we could not estimate timing and location of collisions. Furthermore, these experiments were conducted on healthy participants at rest, clinically PNS and tSCS are used in people with SCI, and in combination with physical activity. It would be beneficial to assess these outcomes during voluntary movement in future work.

## Conclusion

In this study we explored two potential mechanisms by which tSCS may enhance functional recovery: (i) the effects of repeated pairing of descending and afferent volleys at the cord, and; (ii) the immediate effects of afferent input on corticospinal excitability. We have found that repeated pairing of descending and spinal cord stimulation can increase corticospinal excitability when timed to arrive simultaneously at the alpha-motoneurone. Our results suggest that the effect was likely due to an increase in spinal excitability, and can influence functional motor output. We also report that trains of tSCS increased corticospinal excitability, whereas trains of PNS did not, which may explain the immediate recovery of voluntary control, which has been observed in people with chronic SCI when SCS is switched on. We also found that motor thresholds were substantially higher when delivering tSCS in a single HF (10kHz) burst compared with a single pulse of tSCS (square wave), and that short trains of sub-threshold HF tSCS (similar to the intensity delivered in clinical trials incorporating HF-tSCS) facilitated motor evoked potentials, but to a lesser extent than tSCS, delivered at motor threshold.

## Data Availability Statement

The raw data supporting the conclusions of this article will be made available by the authors, without undue reservation.

## Ethics Statement

The studies involving human participants were reviewed and approved by University College London Research Ethics Committee (protocol IDs: 5732/002, 6864/001, and 6864/006). The participants provided their written informed consent to participate in this study.

## Author Contributions

RH, KB, JR, and LD: conception of the work. YA, RH, FL, PB, CK, KB, and LD: data collection. YA, RH, KB, JR, and LD: data analysis and interpretation. YA, RH, and LD: drafting the article. YA, RH, KB, JR, and LD: critical revision of the article. YA, RH, FL, PB, CK, KB, JR, and LD: final approval of the version to be published. All authors contributed to the article and approved the submitted version.

## Conflict of Interest

The authors declare that the research was conducted in the absence of any commercial or financial relationships that could be construed as a potential conflict of interest.

## Publisher’s Note

All claims expressed in this article are solely those of the authors and do not necessarily represent those of their affiliated organizations, or those of the publisher, the editors and the reviewers. Any product that may be evaluated in this article, or claim that may be made by its manufacturer, is not guaranteed or endorsed by the publisher.
